# ﻿Why *Plagiotheciumsylvaticum* (Brid.) Schimp. (*Plagiothecium*, Plagiotheciaceae) has priority over *P.platyphyllum* Mönk.?

**DOI:** 10.3897/phytokeys.241.118858

**Published:** 2024-04-12

**Authors:** Grzegorz J. Wolski, Mikołaj Latoszewski, William R. Buck

**Affiliations:** 1 University of Lodz, Faculty of Biology and Environmental Protection, Department of Geobotany and Plant Ecology, ul. Banacha 12/16, 90–237 Lodz, Poland University of Lodz Lodz Poland; 2 Institute of Systematic Botany, The New York Botanical Garden, Bronx, NY 10458-5126, New York, USA Institute of Systematic Botany, The New York Botanical Garden New York United States of America

**Keywords:** Lectotype, Plagiotheciaceae, *Plagiothecium*, re-assessment, synonymisation, taxonomy, Zennosuke Iwatsuki

## Abstract

Re-assessment of the lectotype of *Hypnumsylvaticum* Brid. (≡ *Plagiotheciumsylvaticum* (Brid.) Schimp.) (B 31 0915 01) showed that this specimen is characterised by dense, 6–10 cm long stems, pale green, yellowish-green to dark green and dull foliage; with complanate, ovate, not imbricate and not julaceous, 2.0–3.0 × 1.0–1.6 mm leaves; acute and denticulate, often eroded apices; 75.0–160.0 × 12.5–20.0 μm laminal cells at mid-leaf, which form diagonal rows, and decurrencies of 3–4 rows of rectangular to square, inflated cells, forming distinct auricules. Thus, this specimen represents the characteristics of the taxon currently referred to as *Plagiotheciumplatyphyllum* Mönk. Taking into account the above and the fact that the name *H.sylvaticum* was published first, the correct name for the species is *Plagiotheciumsylvaticum*. Whereas the later one (*P.platyphyllum*) is a synonym. Additionally, in this article for the name *P.platyphyllum*, a lectotype is designated and a new synonym (Plagiotheciumrutheif.submersum) is proposed for the resurrected *P.sylvaticum*.

## ﻿Introduction

*Hypnumsylvaticum* Brid. [≡ *Plagiotheciumsylvaticum* (Brid.) Schimp.] is one of the oldest names that has been placed in *Plagiothecium* Schimp. (Bridel 1801; Schimper 1851). Importantly, since this taxon was described, its interpretation has changed quite radically. *Hypnumsylvaticum* usually was defined very broadly and many other, often unrelated species were associated with this name (e.g. Gravet (1883); Dixon (1896); Jensen (1939); Koppe (1949); Barkman (1957)). Therefore, dozens of names related to this taxon have been described and also, as indicated Iwatsuki (1970), this species is one of the most complicated in the history of the genus.

**Figure 1.**
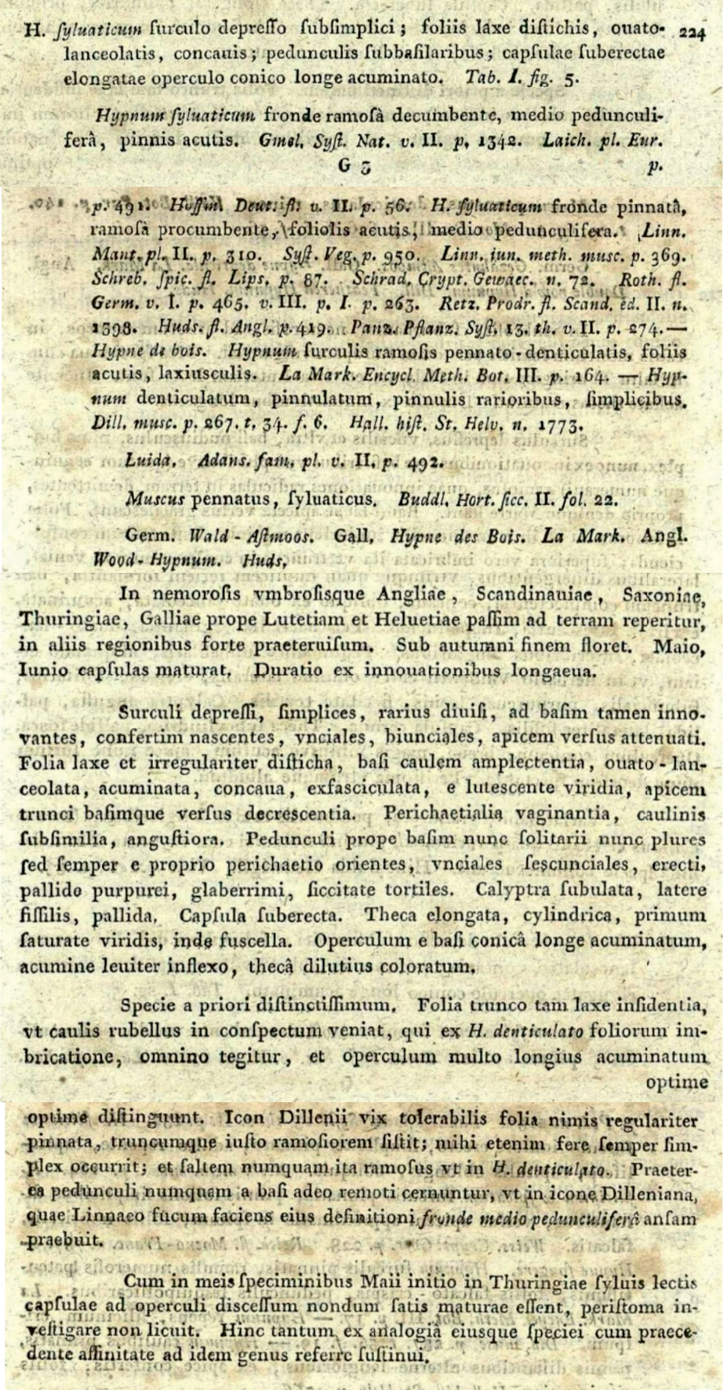
Diagnosis of *Hypnumsylvaticum* from “*Muscologia Recentiorum*” (Bridel 1801).

**Figure 2.**
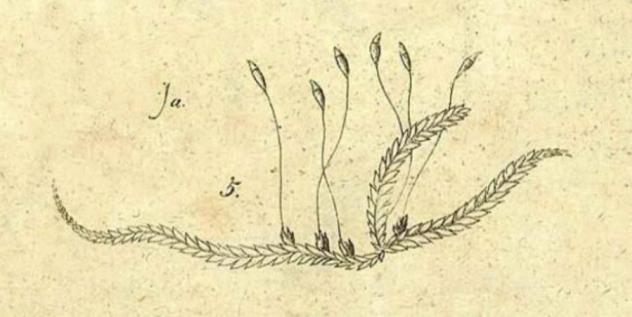
Drawing of *Hypnumsylvaticum* from “*Muscologia Recentiorum*” (Bridel 1801).

**Figure 3.**
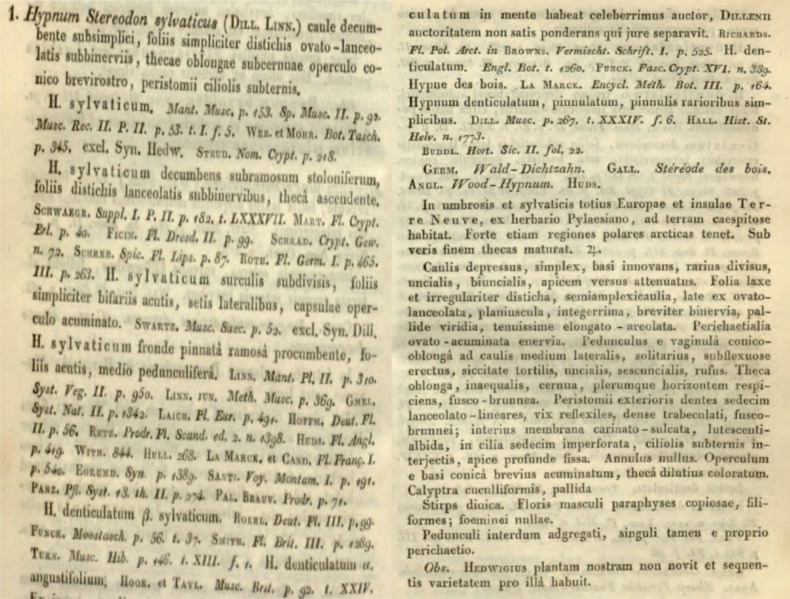
Description of *Stereodonsylvaticus* from “*Bryologia Europaea*” (Bridel 1827).

*Hypnumsylvaticum* was described by Samuel Élisée von Bridel in “*Muscologia Recentiorum*” (Bridel 1801) (Fig. 1) and was also illustrated by the author (Fig. 2). Shortly after it was described, it was not always distinguished as a separate species, but was often treated as a variety, for example, H.denticulatumvar.sylvaticum (Brid.) Turner (Turner 1804). A dozen years later, Bridel in “*Bryologia Universa*” proposed transferring *H.sylvaticum* to the genus *Stereodon* (Brid.) Brid., as *S.sylvaticus* (Brid.) Brid. (Bridel 1827). Then, 24 years later, Wilhelm Philipp Schimper included this taxon in the genus *Plagiothecium*, as *P.sylvaticum* (Brid.) Schimp. (Schimper 1851) and so, until the second half of the 20^th^ century, it was recognised under this name (Fig. 3).

The second half of the 19^th^ century brings a variety of ways of interpreting this taxon. Some authors (e.g. Wilson (1855); Sullivant and Lesquereux (1865); Scheutz (1869)) still included it in the genus *Hypnum* Hedw., giving *P.sylvaticum* as a homotypic synonym. Others, i.e. the vast majority of researchers, following Schimper (1851, 1856), distinguished it as a representative of the genus *Plagiothecium* (e.g. Lorentz (1864); de Notaris (1867); Smith (1870); Jaeger (1875–1876); Schimper (1876); Mitten (1891); Macoun (1892); Husnot (1892–1894).

On the other hand, e.g. Milde (1869), we note one of the first times where this name was recorded as *P.silvaticum* Schimp. in Lindberg [*nom. illeg*. *orthogr*. *pro P.sylvaticum* (Brid.) Schimp.]. Subsequently, *P.silvaticum* would usually appear in the literature as *P.sylvaticum* (e.g. Lindberg (1871, 1879); Molendo (1875); Zetterstedt (1877); Kindberg (1882); Warnstorf (1885); Brotherus and Salen (1890); Klinggraeff (1893); Velenovský (1897); Schiffner (1898)). Another error is an entry given by Macoun (1898), who had a typographical error as *P.sylraticum nom*. *illeg*. *orthogr*. *pro P.sylvaticum* (Brid.) Schimp.

In the second half of the 19^th^ century, several dozen taxa were described within *P.sylvaticum*, mainly as varieties, less frequently as forms. The first of them was the one proposed by Schimper (1856) – P.sylvaticumvar.orthocladium (Schimp.) Schimp., which was a new combination of the previously described *P.orthocladium* Schimp. Currently, this taxon is considered a synonym of *P.cavifolium* (Brid.) Z.Iwats. (Wolski et al. 2021).

A few years later, Molendo (1865, 1866) proposed three varieties for *P.sylvaticum* – P.sylvaticumvar.laxum Molendo, P.sylvaticumvar.myurum Molendo and P.sylvaticumvar.luridum Molendo. Additionally, Walther and Molendo (1868) proposed P.sylvaticumvar.roeseanum (Hampe *ex* Schimp.) A.W.H.Walther & Molendo. Almost at the time when Walther and Molendo (1868) proposed this new variety, Lindberg (1865) wrote the name incorrectly as var. roesei – P.sylvaticumvar.roesei Lindb., with Kindberg (1883) changing its status to a subspecies – P.sylvaticumsubsp.roesei (Lindb.) Kindb. (*orthogr. pro P.roeseanum* Hampe *ex* Schimp.). The reproduction of this error in literature led to its dissemination amongst researchers for decades to come (e.g. Lindberg (1865); Kindberg (1883)).

At the end of the 19^th^ century, the above-mentioned Nils Conrad Kindberg in Macoun (1890) proposed Plagiotheciumsylvaticumvar.squarrosum Kindb. and Limpricht (1897) published two forms – P.sylvaticumf.propaguliferum Ruthe *ex* Limpr. and P.sylvaticumf.elatum Breidl. *ex* Limpr. which, a few years later, Warnstorf (1899, 1905) changed the status to varieties – P.sylvaticumvar.propaguliferum (Ruthe *ex* Limpr.) Warnst. and P.sylvaticumvar.elatum (Breidl. *ex* Limpr.) Warnst. The above-mentioned Carl Warnstorf (1899) at the same time described two other taxa – P.sylvaticumvar.flavescens Warnst. and P.sylvaticumvar.longifolium Warnst.

Additionally, Spruce (1880), at the end of the 19^th^ century, proposed new varieties of the described taxon – P.sylvaticumvar.phyllorhizans Spruce and P.sylvaticumvar.succulentum (Wilson) Spruce. However, for the last one, several years later, Amann and Meylan (1918) proposed a new combination – P.sylvaticumsubsp.succulentum (Wilson) J.J.Amann & Meyl.

Almost at the same time as Spruce (1880), another researcher, Röll (1891, 1915), proposed five infraspecific taxa of this species – P.sylvaticumvar.gracile Röll, P.sylvaticumvar.latifolium Röll *nom. illeg.*, P.sylvaticumvar.submersum Röll, P.sylvaticumf.viride Röll and P.sylvaticumvar.robustum Röll. The latter name (P.sylvaticumvar.robustum) was later illegitimately used by Podpěra (1906) – P.sylvaticumvar.robustum Schiffn. *ex* Podp. *nom. illeg*.

Apart from the above-mentioned bryologists, in the mid-19^th^ century, many researchers published new names, mainly varieties of *P.sylvaticum*: Sendtner (1861) proposed P.sylvaticumvar.connivens Sendtn. in G.Gerber *nom. nud.*; Juratzka (1864) – P.sylvaticumvar.cavifolium Jur. in Rabenhorst; and Gravet (1883) – P.sylvaticumvar.rupestre Warnst. *ex* Grav.

The following years brought additional new varieties, Delogne (1885) proposed P.sylvaticumvar.repens Delogne, Cardot (1885) – P.sylvaticumvar.rivulare Debat *ex* Cardot, Renauld and Cardot (1893) proposed P.sylvaticumvar.sullivantiae (Schimp. *ex* Sull.) Renauld & Cardot, which was a new combination of the previously described *H.sullivantiae* Schimp. *ex* Sull. Another taxon – P.sylvaticumvar.nervosum Renauld is given by Ferdinand François Gabriel Renauld (1894) and Velenovský (1897) published P.sylvaticumvar.orthocarpum Velen. In the same year, Breidler (1897) published new names – P.sylvaticumvar.monoicum Breidl. in Limpricht *nom. nud.*, Limpricht (1897) proposed P.sylvaticumf.robustum Pfeff. *ex* Limpricht *nom. illeg.* and P.sylvaticumf.propaguliferum Lindb., Schiffner (1898) offered P.sylvaticumvar.fontanum Schiffn., while Paris (1898) recognised a new variety – P.sylvaticumvar.nemorale (Mitt.) Paris.

The first half of the 20^th^ century also abounds with dozens of new taxa, mainly varieties of *P.sylvaticum*. At the very beginning of 20^th^ century, Paul Sydow (1904) proposed a new combination of P.sylvaticumvar.cryptarum (Renauld & Hérib.) P.Syd. for a taxon previously classified by Renauld and Héribaud (1899) as P.denticulatumvar.cryptarum Renauld & Hérib. At about the same time, Schiffner (1905) described P.sylvaticumvar.pseudoneckeroideum Schiffn., while Kern (1906) described P.sylvaticumvar.auritum Kern, Bottin (1907) proposed P.sylvaticumvar.minus Bott. and Broeck (1914) published P.sylvaticumvar.filiforme Broeck.

The following years brought even more new names: Cardot (1912) proposed three varieties – P.sylvaticumvar.latifolium Cardot, P.sylvaticumvar.pseudoroeseanum Cardot and P.sylvaticumvar.rhynchostegioides Cardot; Mönkemeyer (1909, 1927, 1949) listed three new taxa, one variety – P.sylvaticumvar.longicuspis Mönk. in Geheeb and two forms – P.sylvaticumf.pungens Mönk. and P.sylvaticumf.acutum Mönk. *nom. inval*.

Subsequent researchers gave further names; Fritz Koppe (1931) distinguished P.sylvaticumvar.neglectum (Mönk.) F.Koppe and P.sylvaticumvar.platyphyllum (Mönk.) F.Koppe., the same author (Koppe 1949) also proposed a new form P.sylvaticumf.laticuspis F.Koppe. Jensen (1939) published two others – P.sylvaticumf.longifolium (Mönk.) C.E.O.Jensen, which is a new combination of P.succulentumvar.longifolium Mönk. described by Mönkemeyer (1927) and P.sylvaticumf.cavernarum C.E.O.Jensen. However, 15 years later, Podpěra (1954) published the same name – P.sylvaticumf.cavernarum Podp. *nom. nud. et nom. illeg*.

In the second half of the 20^th^ century, few authors recognised this taxon. In most cases, it was replaced by *P.neglectum* described by Mönkemeyer (1927). However, Podpěra (1954) proposed P.sylvaticumvar.fluitans Podp. *nom. nud.* and the above-mentioned form P.sylvaticumf.cavernarum Podp. Barkman (1957) reported P.sylvaticumvar.neglectumf.orthocladium (Schimp.) Barkman and P.sylvaticumvar.neglectumf.platyphyllum (Mönk.) Barkman. The last name was given by Landwehr (1966) – P.sylvaticumf.gemmascens Landwehr *nom. nud*.

As the above historical review indicates, over the decades, not only dozens of infraspecific names have been described within *P.sylvaticum*, but also the way of understanding and perceiving this taxon has been very diverse, most often too broadly. Already at the end of the 19^th^ century, Walther and Molendo (1868) distinguished P.sylvaticumvar.roeseanum, which was a new combination of the previously described *P.roeseanum* Hampe *ex* Schimp. now known as *P.cavifolium* (Brid.) Z.Iwats. The idea of combining this taxon with *P.sylvaticum* persisted until the mid-20^th^ century (Molendo 1875; Braithwaite 1896–1905; Héribaud 1899; Meylan 1905; Jensen 1939).

Spruce (1880) described P.sylvaticumvar.succulentum and Amann and Meylan (1918) proposed a new combination of this taxon – P.sylvaticumsubsp.succulentum, indicating its relationship with Hypnumdenticulatumvar.succulentum Wilson, described by Wilson (1855) and currently understood as *P.succulentum* (Wilson) Lindb. This approach was also adopted by, for example, Dixon (1896, 1904).

Paris (1898) distinguished P.sylvaticumvar.nemorale, listing this variety as a new combination of *Stereodonnemoralis* Mitt. now known as *P.nemorale* (Mitt.) A.Jaeger. Koppe (1931) distinguished P.sylvaticumvar.neglectum, reducing *P.neglectum* (currently a synonym of *P.nemorale* (Wolski et al. 2021)) to a variety. The idea of combining this taxon with *P.sylvaticum* appeared in literature until the mid-20^th^ century (Barkman 1957).

The above-mentioned Koppe (1931) distinguished P.sylvaticumvar.platyphyllum, while Barkman (1957) recognised P.sylvaticumvar.neglectumf.platyphyllum, which are new combinations of the previously described *P.platyphyllum* Mönk. (Mönkemeyer 1927).

The consequence of the appearance of new names to describe the same taxon was chaos in its interpretation. In the mid-20^th^ century, Greene (1957) indicated that the use of the name *P.neglectum* should be reconsidered and to replace it with *P.sylvaticum*. Greene’s perception of *P.sylvaticum* is closely related to the taxon currently understood as *P.nemorale*, as indicated by the figures in the text of the manuscript.

The approach presented by Greene (1957) was supported by Nyholm (1965), which gave *P.sylvaticum* (≡ *H.sylvaticum*), for which *P.neglectum* (= *P.nemorale*) is a synonym. Thus, in Europe, some researchers called the same species *P.sylvaticum* (e.g. Greene (1957); Nyholm (1965)), while others called it *P.neglectum* (Podpěra 1954; Barkman 1957).

Outside of Europe, the history of the described taxon is equally dynamic. The first records of *P.sylvaticum* for North America were given by Lesquereux and James (1884), Renauld and Cardot (1892), Macoun (1898), and Cardot and Thériot (1902). However, Robert R. Ireland (1969), in his revision of the genus *Plagiothecium*, excluded *P.sylvaticum* and other related species (P.sylvaticumvar.orthocladium, P.sylvaticumvar.succulentum and *P.neglectum*) from the North American bryoflora. Moreover, he indicated that all North American taxa, so far called *P.sylvaticum*, belong to the *P.roeseanum* complex (= *P.cavifolium* complex).

However, in Japan, *P.sylvaticum* was noted until the mid-20^th^ century (Sakurai 1954). A breakthrough moment in the understanding and perception of this name, not only in Japan, occurred with the Iwatsuki (1970) publication.

Taking into account the above, the aim of the following article is: analysis of the lectotype of *H.sylvaticum* Brid.; determining the taxonomic status of *P.sylvaticum*; and indicating any new synonymy for the examined taxon.

## ﻿Materials and methods

The following research was based on the analysis of the lectotype of *Hypnumsylvaticum* Brid. (B 31 0915 01) which is currently stored in the Botanischer Garten und Botanisches Museum, Freie Universität Berlin, Herbarium B.

Nomenclatural types, original collections and the Wilhelm Mönkemeyer Herbarium are deposited in the Herbarium of the University of Hamburg, Herbarium HBG. This is indicated not only by the Index of Botanists (https://kiki.huh.harvard.edu, accessed 06 November 2023), but also in the Walther and Martienssen (1976) manuscript, which documents the bryological collections of this Herbarium. Specimens of *P.platyphyllum* from the Wilhelm Mönkemeyer Herbarium were borrowed and then subjected to a detailed review and re-assessment.

Additionally, types, original collections and specimens of P.rutheif.submersum Bizot *in sched.* (PC 0132598; PC 0132599) deposited in the Muséum National d’Histoire Naturelle, PC Herbarium were analysed.

## ﻿Results and discussion

### ﻿*Hypnumsylvaticum* Brid. case

**Figure 4.**
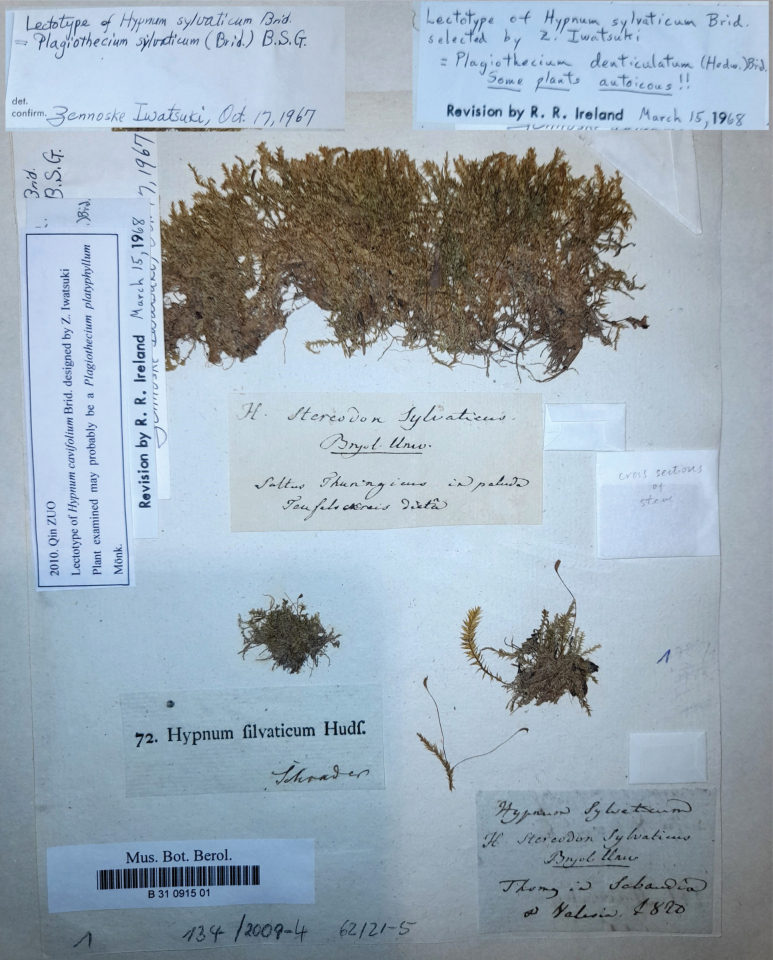
The lectotype (the clump at the top of the sheet) of *Hypnumsylvaticum* (B 31 0915 01).

In 1967, Zennosuke Iwatsuki studied the original collection of *Hypnumsylvaticum* Brid. (B 31 0915 01) [≡ *P.sylvaticum*] (Iwatsuki 1967, in adnot.). He described this specimen as “Lectotype of *Hypnumsylvaticum* Brid. = *Plagiotheciumsylvaticum* (Brid.) B.S.G.” and he used these analyses in his revision of the genus *Plagiothecium* (Iwatsuki 1970) where he indicated that specimen (B 31 0915 01) as the lectotype of *H.sylvaticum* (Fig. 4).

In this revision, Iwatsuki (1970) additionally pointed out that “this specimen has an autoicous inflorescence and a fairly wide-decurrent wing on the leaf” and that “it is identical with plants which we now generally call *P.denticulatum*”. Thus, in the above-mentioned manuscript, Iwatsuki (1970) synonymised *H.sylvaticum* with *P.denticulatum*. Additionally, Iwatsuki wrote that, since the lectotype of *H.sylvaticum* is identical with *P.denticulatum*, “we should use another name for the taxon which has been called “*P.sylvaticum*” or “*P.neglectum*”. Thus, he proposed to use a different, earlier name for these taxa: *P.nemorale* (Mitt.) A.Jaeger.

The above assumption, given by Iwatsuki (1970) that *H.sylvaticum* s.str. is a synonym of *P.denticulatum*, while *P.sylvaticum* sensu auct. and *P.neglectum* are synonyms of *P.nemorale*, has been widely accepted by many bryologists and persists to this day.

After Iwatsuki (1967, in adnot.), the above-mentioned specimen (B 31 0915 01) was analysed by Ireland (1968, in adnot.), who indicated, just the same as Iwatsuki, that this specimen represented *P.denticulatum*, leaving a note on it “Lectotype of *Hypnumsylvaticum* Brid. Selected by Z. Iwatsuki = *Plagiotheciumdenticulatum* (Hedw.) Brid. Some plants autoicous!!” This material was last examined by Zuo (2010, in adnot.), who indicated that “plant examined may probably be a *Plagiotheciumplatyphyllum* Mönk.” (Fig. 4).

**Figure 5.**
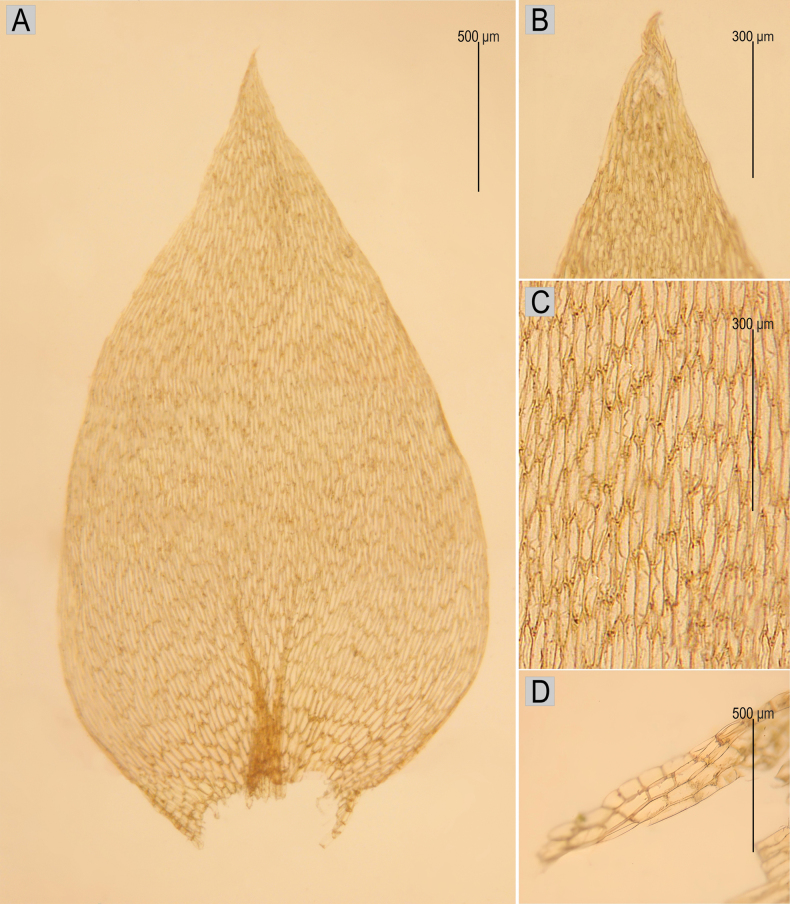
The most important taxonomic features of *Hypnumsylvaticum***A** leaf **B** eroded, denticulate leaf tip **C** cells of the central part of the leaf arranged in regular diagonal rows **D** decurrency composed of inflated cells (based on lectotype B 31 0915 01).

The specimen (B 31 0915 01) representing the lectotype of *H.sylvaticum* (≡ *P.sylvaticum*) is medium size to large, with 6–10 cm long stems; the foliage is pale green, yellowish-green to dark green, dull, without metallic lustre; the plants form rather dense mats; stems are complanate-foliate, in cross-section rounded, 400–450 μm; leaves are complanate, symmetric, ovate, not imbricate and not julaceous; those leaves from the middle of the stem are 2.0–3.0 mm long and the width measured at the widest point is 1.0–1.6 mm; apex is acute and denticulate, often eroded; costae are two, rather thick and strong, extending usually to 1/3 or 1/2 of the leaf length; leaf cells are almost symmetrical, forming diagonal rows, the length and width are variable, but dependent on location: 80–148 × 10–19 μm at the apex, 75–160 × 12.5–20 μm at mid-leaf, 88–112 × 15 μm towards insertion; due to wide cells, the leaf areolation is lax; decurrencies are formed of 3–4 rows of rectangular, inflated cells, forming distinct and long auricules, 0.4–1.1 mm; sporophytes have setae to 4 cm long; capsules are inclined, 2.5 mm long and 1.2 mm wide; operculum is 500 μm long (Fig. 5).

Our critical re-examination of the lectotype specimen of *H.sylvaticum* confirmed Zuo’s suspicions and showed that the above-mentioned specimen (B 31 0915 01) represents the taxon currently understood as *P.platyphyllum* (Fig. 5). The features of the examined specimen, lectotype of *Hypnumsylvaticum* [≡ *P.sylvaticum*] (B 31 0915 01) not only perfectly reflect the features of this species given so far by many researchers (e.g. Jensen (1939); Jedlička (1948); Greene (1957); Nyholm (1965); Smith (2001); Li and Ireland (2008); Cano et al. (2018), but also match perfectly with the type collection of *P.platyphyllum* from the Wilhelm Mönkemeyer Herbarium, currently deposited in HBG.

Thus, taking into account the above facts and the fact that the name *Hypnumsylvaticum* (=*Plagiotheciumsylvaticum*) was published first (Principle III; article 11, Shenzhen Code, Turland et al. 2018) for specimens with dense, 6–10 cm long stems, pale green, yellowish-green to dark green and dull foliage; with leaves complanate, ovate, not imbricate and not julaceous, 2.0–3.0 × 1.0–1.6 mm; acute and denticulate, often eroded apex; 75–160 × 12.5–20 μm cells at mid-leaf, forming diagonal rows and decurrencies of 3–4 rows of rectangular to square, inflated cells, forming distinct auricules, we propose to use the earlier name – *Plagiotheciumsylvaticum* (Brid.) Schimp. and *Plagiotheciumplatyphyllum* should be treated as its synonym.

### ﻿Plagiotheciumrutheif.submersum Bizot *in sched.* case

**Figure 6.**
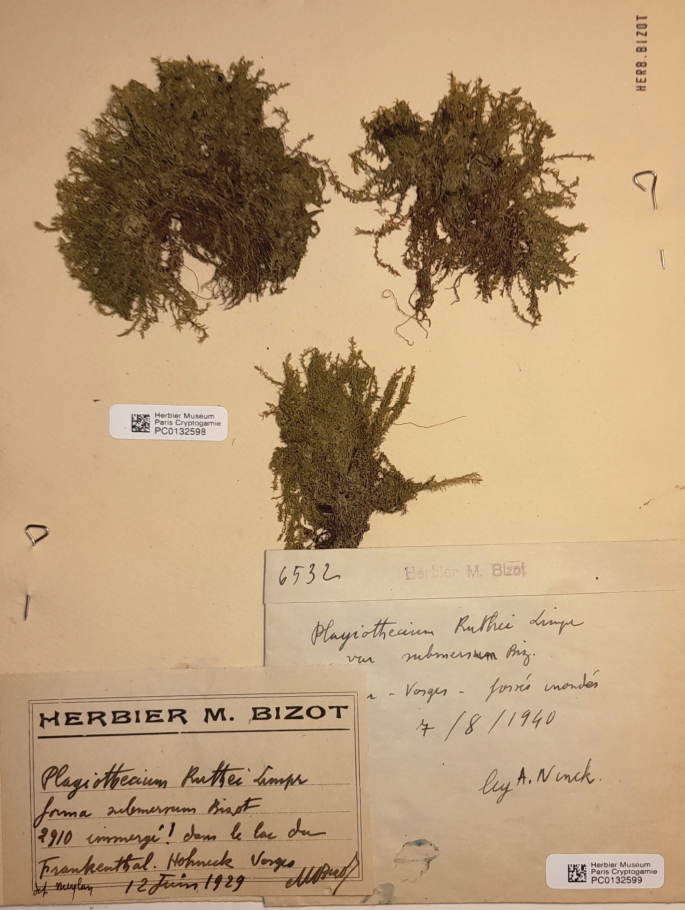
The specimens of Plagiotheciumrutheif.submersum from Muséum National d’Histoire Naturelle (PC0132598).

Plagiotheciumrutheif.submersum (PC0132598), currently housed in PC, consists of three turfs. On the same sheet, there is another specimen (PC0132599) representing the same taxon, also from the *M. Bizot* Herbarium, but collected much later – in 1940 (Fig. 6).

**Figure 7.**
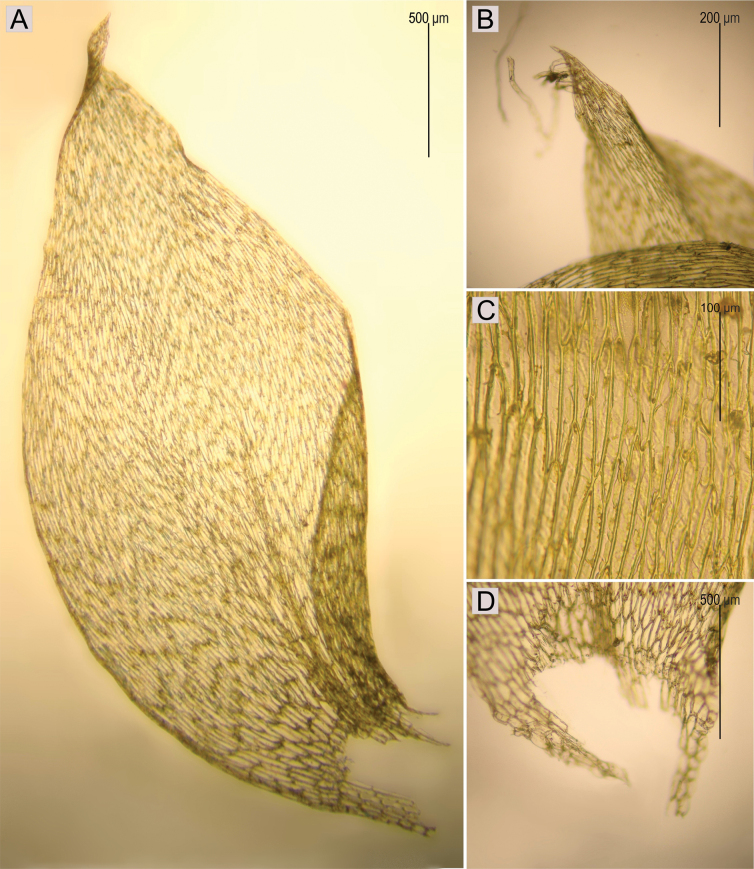
The most important taxonomic features of Plagiotheciumrutheif.submersum**A** leaf shape **B** leaf apex with rhizoids **C** cells of the middle part of the leaf **D** leaf insertion (based on PC0132598).

Plagiotheciumrutheif.submersum is medium size to large; dark green to green; dull; without metallic lustre; forming rather dense mats; stems complanate-foliate, in cross-section rounded; leaves complanate, symmetric, ovate, not imbricate and not julaceous; those leaves from the middle of the stem 2.8–3.0 × 1.0–1.2 mm; apex eroded, acute and denticulate, with commonly occurring rhizoids; costae two, rather thick and strong, extending usually to 1/3 or 1/2 of the leaf length; leaf cells almost symmetrical, these near apex often eroded, the length and width variable, but dependent on location: 85–160 × 9–17.5 μm at the apex, 112.5–150 × 12.5–15 μm at mid-leaf, 46–130 × 19–34.5 μm towards insertion; due to wide cells, leaves areolation lax; decurrencies of 3–4 rows of rectangular to square, inflated cells, forming distinct auricules, 600 μm long; sporophytes unknown (Fig. 7).

Taking into account the above facts and the fact that the specimen representing P.rutheif.submersum is identical to *P.sylvaticum* (= *P.platyphyllum*), we propose the former herbarium name (P.rutheif.submersum) treated as a synonym of the latter (*P.sylvaticum*).

### ﻿Lectotypification of *Plagiotheciumplatyphyllum* Mönk.

*Plagiotheciumplatyphyllum* was described by Wilhelm Mönkemeyer (1927) in “Die Laubmoose Europas”. Mönkemeyer (1927) quotes various specimens that he analysed and on the basis of which he described this taxon. On the other hand, he states that: „In Laubwäldern auf Humus, über Gestein, an Felsen” (in deciduous forests on humus, on rocks); „in der Grundform von mit zuerst bei Gersfeld in der Rohn 1906. Ferner 1911 im Böhmerwalde bei Eisenstein gesammelt (near Gersfeld in the Rohn in 1906. Further collected in 1911 in the Bohemian forest near Eisenstein). „Ferner mir aus Thüringen und dem sächsischen Vogtlande unter anderer Bezeichnung bekannt geworden (…) Aus dem Harze, Thüringen, der Rhön, dem Fichtelgebirge, aus Böhmen, dem Bayerischen Walde, Mahren, der Schweiz (Kanton Uri), Norditalien (Provinz Como) und Bulgarien mir bekannt geworden” (also known to me under a different name from Thuringia and the Saxon Vogtland (…), from the Harz, Thuringia, the Rhön, the Fichtel Mountains, from Bohemia, the Bavarian Forest, Moravia, Switzerland (Canton of Uri), northern Italy (Province of Como) and Bulgaria became known to me).

**Figure 8.**
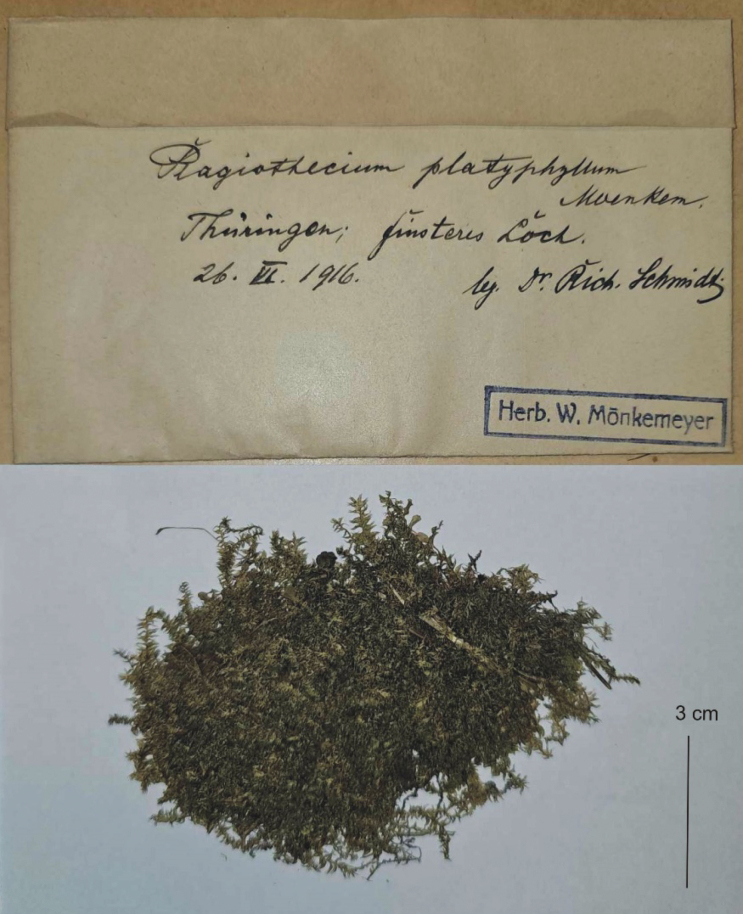
The lectotype of *Plagiotheciumplatyphyllum* Mönk. (HBG).

Analysing the entire Mönkemeyer collection of *Plagiotheciumplatyphyllum* stored in the HBG Herbarium, we could conclude that most of the specimens from those cited by Mönkemeyer in this Herbarium were absent. However, a specimen hand-signed by Mönkemeyer from his private herbarium was found, specimen cited by him as “aus Thüringen”. This specimen is characterised by a large turf with sporophytes material (Fig. 8). Therefore, it was decided to propose this specimen as the lectotype of *Plagiotheciumplatyphyllum* Mönk.

### ﻿Taxonomic treatment

*Plagiotheciumsylvaticum* (Brid.) Schimp., Bryol. Europ. 5: 192, 503 (1851); *Hypnumsylvaticum* Brid., Muscol. Recent. 2(2): 53, 1 f. 5 (1801) (Figs 1, 2); Hypnumdenticulatumvar.sylvaticum (Brid.) Turner, Muscol. Hibern. Spic. 146 (1804); *Stereodonsylvaticus* (Brid.) Brid., Bryol. Univ. 2: 825 (1827); Hypnumdenticulatumsubsp.sylvaticum (Brid.) Boulay, Musc. France, Mousses 85 (1884); Plagiotheciumdenticulatumsubsp.sylvaticum (Brid.) Dixon, Stud. Handb. Brit. Mosses 437 (1896). **Lectotype** (the clump at the top of the sheet, selected by Iwatsuki 1970): [Germany], saltus Thuringicus in paluda, *ex* herb. Brid., B 31 0915 01! (Figs 4, 5).

*Plagiotheciumplatyphyllum* Mönk., Laubm. Europ. 866, 207b (1927); P.sylvaticumvar.platyphyllum (Mönk.) F.Koppe, Abh. Ber. Naturwiss. Abt. Grenzmärk. Ges. Erforsch. Heimat Schneidemühl 1931: 80 (1931); P.neglectumsubsp.platyphyllum (Mönk.) Szafran, Fl. Polsk. Mchy 2: 288 (1961), *comb. inval.* Type: Germany, bei Gersfeld in der Rohn 1906, ferner mir aus Thüringen und dem sächsischen Vogtlande unter anderer Bezeichnung bekannt geworden; The Czech Republic, ferner 1911 im Böhmerwalde bei Eisenstein gesammelt. **Lectotype** (designated here): Germany, Thüringien, Finsteres Loch, 26 June 1916, leg. *Rich. Schmidt*, HBG! syn. nov. (Fig. 8).

Plagiotheciumrutheif.submersum Bizot, *in sched*. Basis: France, Vosges, Hohneck, immergé dans le lac du Frankenthal, *M. Bizot 2910*, PC0132598! syn. nov. (Figs 6, 7).

## ﻿Additional information

### Author ORCIDs

Grzegorz J. Wolski https://orcid.org/0000-0003-1480-8003

Mikołaj Latoszewski https://orcid.org/0009-0003-5228-210X

### Data availability

All of the data that support the findings of this study are available in the main text.
